# A Subject-Specific Acoustic Model of the Upper Airway for Snoring Sounds Generation

**DOI:** 10.1038/srep25730

**Published:** 2016-05-23

**Authors:** Shumit Saha, T. Douglas Bradley, Mahsa Taheri, Zahra Moussavi, Azadeh Yadollahi

**Affiliations:** 1 Department of Biomedical Engineering, University of Manitoba, Winnipeg, Canada; 2 Toronto Rehabilitation Institute-University Health Network, Toronto, Canada; 3 Department of Medicine, University of Toronto, Toronto, Canada; 4 Institute of Biomaterials and Biomedical Engineering, University of Toronto, Toronto, Canada

## Abstract

Monitoring variations in the upper airway narrowing during sleep is invasive and expensive. Since snoring sounds are generated by air turbulence and vibrations of the upper airway due to its narrowing; snoring sounds may be used as a non-invasive technique to assess upper airway narrowing. Our goal was to develop a subject-specific acoustic model of the upper airway to investigate the impacts of upper airway anatomy, e.g. length, wall thickness and cross-sectional area, on snoring sounds features. To have a subject-specific model for snoring generation, we used measurements of the upper airway length, cross-sectional area and wall thickness from every individual to develop the model. To validate the proposed model, in 20 male individuals, intensity and resonant frequencies of modeled snoring sounds were compared with those measured from recorded snoring sounds during sleep. Based on both modeled and measured results, we found the only factor that may positively and significantly contribute to snoring intensity was narrowing in the upper airway. Furthermore, measured resonant frequencies of snoring were inversely correlated with the upper airway length, which is a risk factor for upper airway collapsibility. These results encourage the use of snoring sounds analysis to assess the upper airway anatomy during sleep.

Snoring is present in 20–40% of the general population^1^, and is associated with obesity, nasal obstruction, use of alcohol and cigarettes^2^. Snoring sounds are generated by vibration of pharyngeal tissue due to the narrowing and the consequent increases in the turbulent airflow in the upper airway^3^. Several factors such as increased pharyngeal fat^4^, reduction in pharyngeal dilator muscle tone at sleep onset^4^, and rostral fluid shift from the legs to the neck^5^ may contribute to the upper airway narrowing during sleep. However, the underlying mechanisms of the upper airway narrowing are multifactorial and may change from night to night in the same individual^6^. Consequently, it is difficult to use imaging techniques such as MRI or endoscopy to investigate variations in the upper airway during sleep such as its narrowing, collapsibility, or airway wall thickness^7^.

On the other hand, snoring sounds can be recorded conveniently with a microphone placed on the neck, or in the vicinity of the patient in the room. Snoring can be a sign of obstructive sleep apnea (OSA)^8^; a common respiratory disorder present in approximately 10% of adult population. OSA is characterized by repetitive complete or partial collapse of the upper airway during sleep^4^. Consequently, most of studies on acoustic analysis of snoring sounds focused on the association between OSA severity and a variety of snoring sounds features such as intensity^9^,^10^, power spectral measures^11^,^12^, bi-spectral and non-linear measures^13^, formant frequencies^14^ and temporal features^15^. However, very little emphasis was placed on the effects of variations in the upper airway anatomy during sleep on snoring sounds features. Liu *et al*. used a 3D model of the human head to identify the source of snoring sounds; however, the model was computationally intensive and highly dependent on having an accurate 3D model of the head for every individual^16^. Ng *et al*. developed an electrical equivalent model of the upper airway to investigate the effects of upper airway narrowing on spectral features of snoring sounds^17^. They observed general agreement between the spectral features of simulated and actual recorded snoring sounds in 40 subjects. However, since they did not measure the upper airway cross-sectional area (UA-XSA) in their population, a detailed subject-specific validation of the model to assess the effects of upper airway narrowing on the characteristics of recorded snoring sounds was missing.

To address this gap, we aimed to develop a subject-specific acoustic model of the upper airway for snoring sounds generation. Based on the physics of sound generation in a tube, we hypothesized that snoring sounds intensity will be directly related to narrowing of the upper airway, and the resonant frequency of snoring sounds will be inversely related to the length of upper airway. To have a subject-specific model, we measured the neck circumference (NC), upper airway length (UA-Length) and UA-XSA of human participants and incorporated them in the model for snoring sounds generation. For every individual, the modeling results for snoring sounds intensity and resonant frequencies were compared with those measured from recorded snoring sounds in the same individual during sleep.

## Results

### Baseline characteristics of participants

Twenty men participated in a daytime sleep study and their sleep was assessed with a full in-laboratory polysomnography (PSG) (See methods section for details). Table 1 shows the baseline characteristics of the subjects. Although this was a daytime study, participants slept for an average of 150 minutes (Table 2), and 14 out of 20 had at least one full sleep cycle, including both rapid eye movement (REM) and non-REM sleep stages. Participants spent most of the sleep time in stage 2 of non-REM (N2) sleep (Table 2). The participants had a wide range of OSA severity, as assessed by apnea-hypopnea index (AHI, number of apneas and hypopneas per hour of sleep). Nine subjects had no or mild sleep apnea (AHI < 15), five had moderate sleep apnea (15 ≤ AHI < 30) and six had severe sleep apnea (AHI ≥ 30).

Before and after sleep, while subjects were supine, NC was measured with a measuring tape, and UA-Length and average of UA-XSA (both from velum to glottis) were measured with acoustic pharyngometry (See methods section for details). From before to after sleep, there were significant increases in NC (∆NC: 0.5 ± 0.3 cm, p < 0.001) and decreases in UA-XSA (∆UA-XSA: −0.4 ± 0.3 cm^2^, p < 0.001). As we have shown before, these changes could be due to rostral fluid shift from the legs to the neck while supine^5^. Airway wall thickness which was estimated based on NC and UA-XSA (See methods section for details), increased from before to after sleep (∆: 0.2 ± 0.08 cm, p < 0.001). There was a strong correlation between reductions in UA-XSA and increases in airway wall thickness (r = −0.73, p < 0.001).

### Snoring sounds features

**Figure 1:**
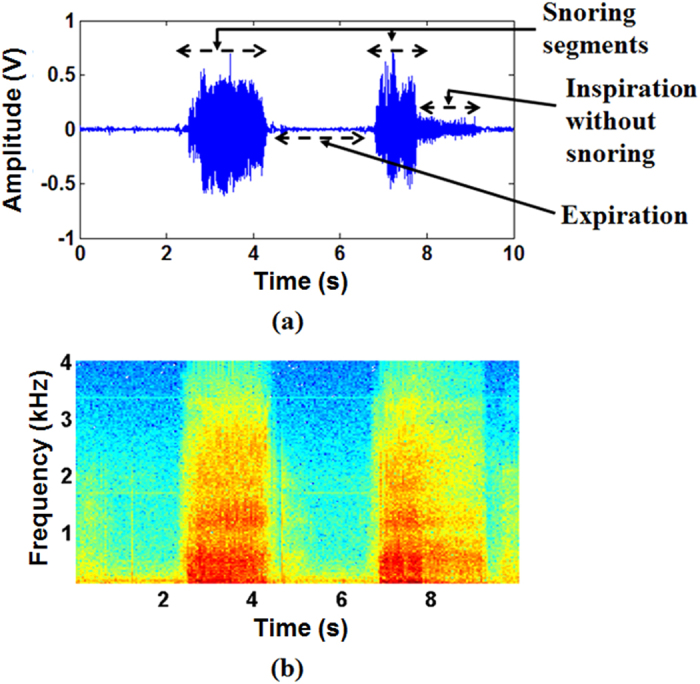
Segmentation of snoring sounds from breath sounds. (**a**) Extracted snoring segments from a 10 seconds recording of breath sounds; (**b**) Spectrogram of the extracted snoring segments as presented in (**a**). In all frequency bands, snoring sounds intensity were larger than those of the inspiratory and expiratory breath sounds without snoring.

Snoring and breath sounds were recorded with a microphone attached to the neck (See methods section for details). Snoring segments were extracted manually by listening to the sounds and observing them in the time and frequency domains. Figure 1 shows a 10-second sample of recorded breath sounds, along with the manual annotation of the snoring segments. While both inspiratory and expiratory snoring were detected, since the snoring mechanisms may be different during inspiration and expiration and inspiratory snoring are more common, only inspiratory snoring were investigated in this study.

**Figure 2:**
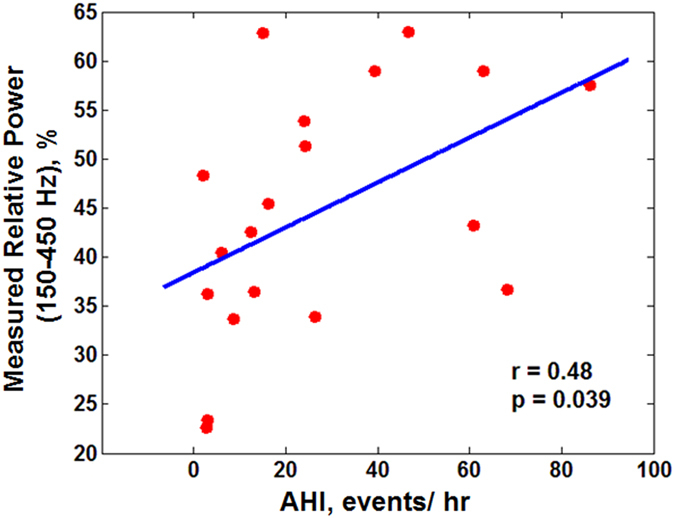
Relationship between apnea-hypopnea index (AHI) and measured relative power of snoring sounds (assessed by Pearson Correlation Coefficient). Increases in AHI were significantly correlated with the increases in the measured relative power of snoring sounds within 150–450 Hz frequency range.

For every individual, 342 ± 223 inspiratory snoring segments were extracted (134.2 ± 96.0 snoring per hour of sleep). The snoring segments were band-pass filtered in the frequency range of 100–4000 Hz to remove the effects of heart sounds in the low frequency range as well as extraneous noises in the high frequency range^18^,^19^. Various features in the temporal and spectral domains were extracted from the snoring sounds (See methods section for details). The total snoring time in each sleep stage normalized by the time spent in each sleep stage (snoring time index) was similar for different sleep stages (p > 0.10), as well as for non-REM vs. REM sleep (p > 0.10). The measured average power of snoring sounds was 38.2 ± 5 dB in the frequency band of 100–4000 Hz. There was a positive correlation between the measured relative power of snoring sounds in the frequency range of 150–450 Hz and the AHI (Fig. 2, r = 0.48, p = 0.039).

### Acoustic model of the upper airway for snoring sounds generation and propagation

**Figure 3:**
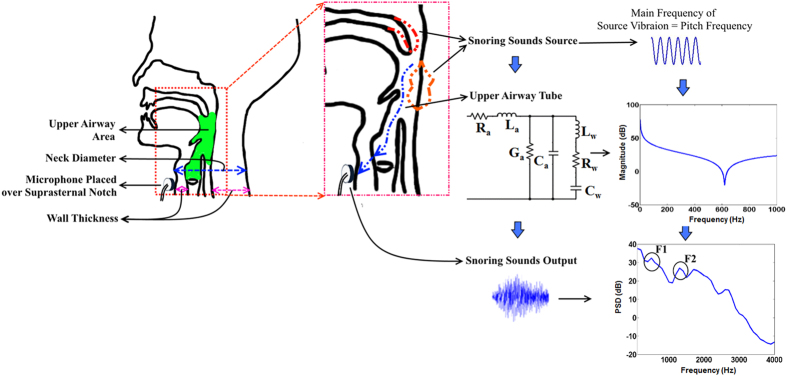
An overview of the proposed block scheme of snoring sounds generation and propagation along with the acoustic model of upper airway. Snoring sounds is generated by the vibration of soft palate or pharyngeal wall, then would propagate through the upper airway and then recorded by the microphone placed over the suprasternal notch. The main frequency of snoring sounds source is analogous to the calculated pitch frequency of snoring sounds. The upper airway was modeled by the electrical equivalent circuit of a collapsible tube with non-rigid wall (R_a_, resistance; L_a_, inertance; C_a_, compliance; G_a_, conductance; L_w_, wall inertance; R_w_, wall resistance; C_w_, wall compliance) using measured upper airway area, neck radius (neck diameter/2), wall thickness showed in the anatomy of upper airway. The power spectral density (PSD) of the snoring segment extracted from recorded data with the marking of formant frequencies is also shown.

Based on the theory of sounds generation in a collapsible tube^20^, and the basics of changes in the oral cavity for vowel articulations^21^, we assumed a simplified two-compartmental model for snoring sounds generation and propagation (Fig. 3). These compartments included, first, the snoring sounds generation within the upper airway, due to vibration of the soft palate and uvula, and/or of the tissues between the soft palate and epiglottis; and second, the entire upper airway, which was modeled as a collapsible tube through which the pressure fluctuations due to snoring are propagated to the microphone located on the suprasternal notch.

We modeled the snoring source vibration as a sinusoid signal with its frequency equal to the measured pitch frequencies of the recorded snoring sounds (See methods section for details). For every individual, the pitch frequency of each snoring segment was estimated and averaged for entire sleep. The mean and standard deviation of the measured pitch frequencies among all subjects was 102.1 ± 20.6 Hz. For the upper airway response, we developed an electrical equivalent model of a collapsible tube^22^,^23^,^24^ (See methods section for details). The parameters of the upper airway model, such as resistance and capacitance, were defined based on UA-Length, UA-XSA and airway wall thickness. To validate the model, resonant frequencies and average power of snoring sounds based on modelling and measured data were estimated and compared.

### Effects of upper airway anatomy on the resonant frequencies of snoring sounds

**Figure 4:**
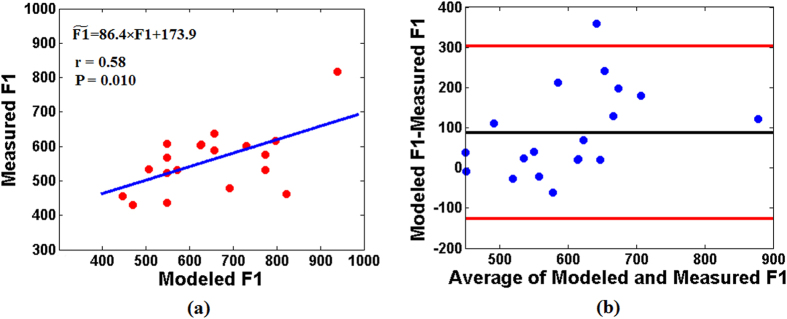
Agreement between modeled and measured F1 of the snoring sounds. (**a**) Scatterplot of the F1 of the recorded snoring sounds (measured F1) and F1 simulated from the upper airway tube model (modeled F1) along with the linear regression equation of modeled and measured F1, where 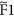 is the measured F1. (**b**) Bland–Altman plot, the black line indicates the average difference and the red lines present the mean ± 1.96 of standard deviation (boundaries of 95% confidence interval) of the difference between modeled and measured F1.

For every subject, resonant frequencies of snoring sounds were calculated as the averages of first and second formants (F1 and F2, respectively) of all recorded snoring sounds for entire sleep duration. The average and standard deviation of measured F1 and F2 among all subjects were 572.1 ± 122.6 Hz and 1626.1 ± 168.7 Hz, respectively. On the other hand, for each subject, we used the baseline measurements of UA-XSA, wall thickness, and UA-Length before sleep to estimate F1 (modeled F1). There was a significant and positive correlation between the modeled and measured F1 (Fig. 4a, r = 0.58, p = 0.01). Also, based on the Bland-Altman analysis (Fig. 4b), there was a good agreement between modeled and measured F1.

#### Results obtained from the modeled snoring frequencies

We calculated the effects of changes in UA-XSA, UA-Length and wall thickness on resonant frequencies of the model. The modelling results showed that length of the upper airway was inversely related to the modeled F1. Increasing the UA-Length from 7.0–12.5 cm, modeled F1 decreased from 800 Hz–450 Hz (r = −0.98, p < 0.001). On the other hand, there were no significant correlations between modeled F1 and variations in the UA-XSA or airway wall thickness (p > 0.05 for both).

#### Results obtained from measured snoring frequencies

**Figure 5:**
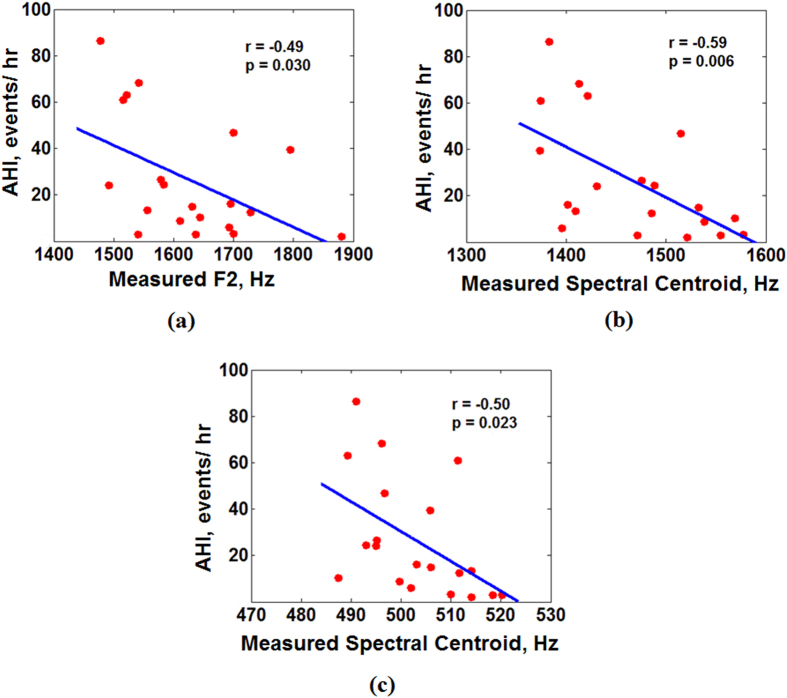
Relationship between apnea-hypopnea index (AHI) and measured snoring sounds frequencies (assessed by Pearson Correlation Coefficient). (**a**) Decreases in the measured F2 (calculated over entire sleep duration) were significantly correlated with the increases of AHI; (**b**) Decreases in the measured spectral centroid of snoring sounds (calculated over the entire sleep duration) within 1200–1800 Hz frequency range were significantly correlated with the increases of AHI; (**c**) Decreases in the measured spectral centroid of snoring sounds (calculated over the entire sleep duration) within 450–600 Hz frequency range were significantly correlated with the increases of AHI.

Previous studies have shown that UA-Length is longer in patients with OSA than in those without OSA^25^,^26^. Based on recorded snoring sounds, there were significant and negative correlations between AHI and measured F2 (Fig. 5a, r = −0.49, p = 0.030), AHI and measured spectral centroid of snoring sounds in the frequency range of 1200–1800 Hz (associated with F2^27^) (Fig. 5b, r = −0.59, p = 0.006), and AHI and measured spectral centroid in 450–600 Hz (associated with F1^27^) (Fig. 5c, r = −0.50, p = 0.023).

Therefore, both modelling and measured results suggested that there was an inverse relationship between resonant frequencies of the snoring sounds and UA-Length.

### Effects of upper airway anatomy on the intensity of snoring sounds

We calculated the effects of changes in UA-XSA, UA-Length, and airway wall thickness on the snoring sounds intensity.

#### Results obtained from the modeled snoring intensity

**Figure 6:**
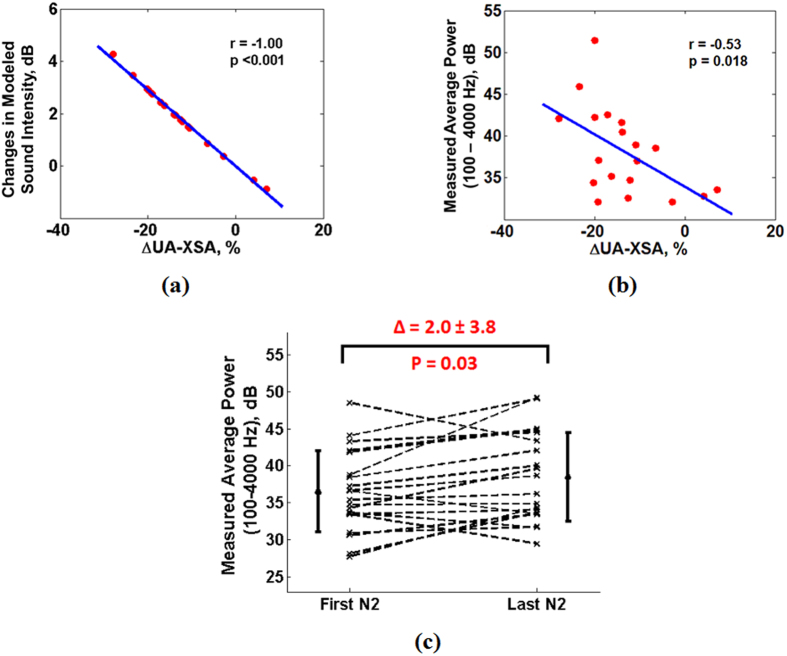
Relationship between changes in upper airway cross sectional area (UA-XSA) during sleep and snoring sounds intensity (assessed by Pearson Correlation Coefficients). (**a**) Result obtained from the modeled snoring intensity: narrowing in the UA-XSA was strongly correlated with increases in the intensity of the sound; (**b**) Result obtained from the measured snoring intensity: narrowing in the UA-XSA during sleep was significantly correlated with increases in the measured average power of snoring sounds (calculated over the entire sleep duration) within 100–4000 Hz frequency range; (**c**) Results obtained from the measured snoring intensity: there were significant increases in the measured average power snoring sounds from first part to the last part of the N2 sleep stage (assessed by paired t-test).

There was a significant correlation between percentage narrowing in the UA-XSA from before to after sleep and the gain of the upper airway model (Fig. 6a, r = −1.00, p < 0.001); indicating that upper airway narrowing increases the modeled intensity of snoring. On the other hand, there were no significant correlations between the gain of the upper airway model and changes in wall thickness or UA-Length (p > 0.10 for both).

#### Results obtained from measured snoring intensity

There was a significant correlation between the measured average intensity (average power of recorded snoring sounds in 100–4000 Hz) of all snoring segments during sleep and the percentage narrowing of UA-XSA from before to after sleep (Fig. 6b, r = −0.53, p = 0.018). Moreover, for each subject, we calculated the average intensity of 5 snoring segments in the first and last 15 minutes of N2 sleep. The results showed that from the first to the last part of N2 sleep, there was a significant increase in the measured intensity (Fig. 6c, ∆ = 2.0 ± 3.8 dB). These were similar to those based on the simulation (p = 0.947). On the other hand, there were no significant correlations between the measured intensity of recorded snoring sounds and changes in the upper airway wall thickness or UA-Length (p > 0.1 for both).

Therefore, both modelling and measured results suggested that there was an inverse relationship between the snoring sounds intensity and UA-XSA.

## Discussion

To the best of our knowledge, this is the first study to develop a subject-specific model of the upper airway and to consider the fact that changing in the upper airway area could also change other anatomical factors of the upper airway such as wall thickness. Our model showed that the upper airway narrowing during sleep increases snoring intensity, while increases in the UA-Length reduced the resonant frequency of snoring sounds. The model was validated against recordings of actual snoring. Furthermore, we found that measured resonant frequencies of snoring sounds were inversely related to the OSA severity.

Our proposed model for the snoring sounds generation included snoring source vibration and propagation of the vibrations through the upper airway. A novelty of this study was that we modeled the snoring source as a single frequency vibratory signal due to either soft palate vibration or pharyngeal tissue vibration at the site of upper airway narrowing. Similar to speech articulation^21^, we considered the measured pitch frequency of snoring sounds as the main vibrating frequency of snoring source^1^. We found the measured pitch frequency of the recorded snoring sounds were less than 150 Hz. Previous studies in dog models for inducing snoring have shown that the main frequency of snoring sounds was in the range of 64–135 Hz^28^,^29^. Although, dog models are not true representatives of human upper airway, these results support our assumption that pitch frequency can represent the main vibratory source of snoring sounds. As part of having a subject-specific model, for every individual we used the measured pitch frequency of his recorded snoring to characterize the snoring sounds source.

An important finding of this study was that narrowing of the upper airway during sleep may increase the turbulent airflow and increase the snoring sounds power. On the other hand, narrowing in the upper airway can increase sleep apnea severity^30^. Our results showed a positive correlation between AHI and measured relative power of snoring sounds. These results are consistent with those of Maimon *et al*. who showed that snoring sounds intensity is related to severity of OSA^9^.

Another major finding of our model was that modeled resonant frequency of snoring sounds was inversely related to the upper airway length. Conversely, there was an inverse relationship between OSA severity and measured resonant frequency of snoring sounds. Imaging and cephalometric studies have shown that patients with OSA have longer upper airway than those without OSA^4^,^25^,^26^. Similarly, previous studies have shown that in vowel articulation, formant frequencies for articulating /i/ or /u/ (e.g. in “see” or “moo”) are significantly lower in patients with OSA than non-OSA individuals^31^,^32^. Although, snoring sounds and speech have different vibratory sources, the role of upper airway in generating sounds may be similar for both signals. Therefore, these results may provide proof of concept for the potential applications of resonant frequencies of snoring sounds to assess the effects of upper airway length on OSA severity.

Previous studies have shown that the main frequencies of the snoring sounds vary depending on the site of obstruction^12^,^33^. While the main frequencies of snoring due to the narrowing at the base of tongue were above 650 Hz, frequencies of palatal snoring were below 450 Hz^12^,^33^. In this study, we did not have any measurement to assess the site of upper airway obstruction. However, if we assume that the length of upper airway in our model represents the distance between the site of obstruction and the recording microphone, the resonant frequency of simulated palatal snoring was 450 Hz; while it was approximately 800 Hz for the snoring originating closer to the glottis. These simulation results, which should be verified in future studies, may provide non-invasive techniques to assess the site of upper airway obstruction in snorers and patients with OSA.

Our study was subject to some limitations. Since daytime sleep studies were performed, these results may not be the same as those during nocturnal sleep. Furthermore, we modeled the snoring sounds generation and propagation using a single segment tube model. Further studies will be needed to develop a multi-segment tubular model to include the effects of oral and nasal cavities on snoring sounds features. Also, participants in this study were non-obese men. Future research will need to examine snoring features and upper airway properties in other populations such as obese individuals, women, and children. Furthermore, we limited the participants to supine sleep. Therefore, our results may not be fully applicable to other sleeping postures.

In conclusion, this study attempted to develop a subject-specific acoustic model of the upper airway for snoring sounds generation and to investigate the effects of changes in upper airway narrowing and length on snoring sounds features. Future studies should validate the proposed model based on simultaneous recordings of snoring sounds, UA-XSA and sites of collapse during sleep on a larger population. Since snoring sounds can be recorded non-invasively and conveniently during multiple nights; once validated, the proposed model may be used as a tool to assess the dynamics of upper airway narrowing during sleep.

## Methods

### Participants

Healthy non-obese and normotensive men were recruited by advertisement and participated in this study^34^. Individuals with a history of cardiovascular, respiratory, renal, or neurological diseases, or previously diagnosed of OSA, or those who slept less than one hour during the protocol, or with central dominant sleep apnea were excluded from the study.

### Experimental protocol

Participants arrived in the sleep laboratory at early afternoon after a night of voluntary sleep deprivation and were instrumented for sleep studies. Baseline measurements including UA-XSA, UA-Length, and NC were performed in supine position before sleep and just after the participants woke up. Breath and snoring sounds were recorded continuously and simultaneously with polysomnography during sleep^34^.

The experimental protocol was approved by the Research Ethics Board of Toronto Rehabilitation Institute-University Health Network and all participants signed written consent prior to participation^34^. The study was performed in accordance with the approved guidelines and regulations.

### Data measurement

#### Sleep studies

Daytime polysomnography was performed for the convenience of participants and the research personnel. Thoracoabdominal motion, nasal pressure, and arterial oxyhemoglobin saturation (SaO_2_) were monitored by respiratory inductance plethysmography, nasal cannulae, and oximetry, respectively^35^. Scoring sleep stages and arousals were done by a specialist using standard techniques and criteria. The definition and classification of apneas (cessation of airflow to the lungs for at least 10 s) and hypopneas (>50% decrease in breathing airflow for more than 10 s with blood oxygen desaturation of >3%) were done in accordance the American Academy of Sleep Medicine (AASM)^36^. To minimize any possible effect of postural changes on AHI and other variables, participants slept supine on a single pillow for the entire study duration. Sleep studies were scored by personnel blind to the fluid measurements and vice versa^34^.

#### UA-XSA, UA-Length and NC measurement

UA-XSA and UA-Length (the distance from velum to glottis) were measured by acoustic pharyngometry^37^. NC was assessed by a measuring tape. A line was drawn just above the cricothyroid cartilage to ensure the measurements before after sleep were made at the same level.

#### Breath and snoring sounds recoding

Breath along with snoring sounds were recorded by a Sony EMC-44B omni-directional microphone. The microphone was placed over suprasternal notch using double-sided adhesive tape. The sounds were filtered by a low-pass filter (cut off frequency of 5 kHz) using Biopac DA100C, and digitized at sampling rate of 12.5 kHz using MP150 Biopac System^38^.

### Data analysis

After manual segmentation of snoring sounds, several features in the temporal and spectral domains were extracted.

- Temporal Feature:

- Snoring Time Index: Total snoring time in each sleep stage divided by the time spent in each sleep stage.

- Spectral Features: Snoring segments were band-pass filtered in the frequency range of 100–4000 Hz to remove the effects of heart sounds in low frequency ranges as well as high frequency noises^18^. Power spectral density (PSD) of each snoring segment was calculated based on the Welch method with a Hamming window of 100 ms and 50% overlap between adjacent windows. Based on the PSD, following features were calculated:

- Average Power: Average power of the snoring sounds in eight frequency bands of 100–4000 Hz, 100–150 Hz, 150–450 Hz, 450–600 Hz, 600–1200 Hz, 1200–1800 Hz, 1800–2500 Hz, and 2500–4000 Hz were calculated^39^ (See Supplementary File S1).
- Relative Power: The average power of snoring sounds in each sub-band divided by the average power in entire frequency band (100–4000 Hz).
- Spectral Centroid: It represents the frequency that contains the maximum power of snoring sounds in any of the eight frequency bands^40^,^41^.

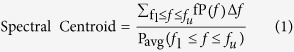


Here, P_avg_ = Average power; *f*_*1*_ = Lower band frequency; *f*_*u*_ = Higher band frequency; *P(f) *= Estimated power spectral density.

- Pitch and Formant Frequencies: Pitch frequency was calculated based on the robust algorithm for pitch tracking^42^. For calculating formants, snoring segments were pre-processed using a Hamming window (window size of 20 ms) and a pre-emphasizing filter. Then, 16^th^ order linear predictive coding (LPC) spectrum of the snoring sounds was estimated to extract formants^43^,^44^. Pitch was calculated using the validated “Voicebox” toolbox^45^.

### Modelling of the upper airway for snoring sounds generation and propagation

We proposed a simplified two-compartmental model for snoring sounds generation that included snoring source vibration and the upper airway response as a collapsible tube (Fig. 3).

#### Snoring source vibration

Snoring sounds can be generated either by oscillation of the soft palate or the upper airway wall^29^. Based on the Bernoulli’s theorem, in a collapsible tube such as the upper airway, upper airway narrowing or increased negative intra-thoracic pressure during inspiration increases the airflow speed which will cause a pressure drop across the upper airway; this sequence of events will further increase the negative pressure in the upper airway, narrow the upper airway and increase the airflow speed^3^. The consequence of these events is an increase in turbulence of airflow within the upper airway, which leads to vibration of soft palate or the airway wall tissue and induces snoring sounds^3^. Considering speech articulation, vibration of soft palate or the upper airway wall during snoring sounds generation can be assumed to be analogous to the vibration of vocal cord^1^. Therefore, we assumed that pitch frequency represent the fundamental frequency of snoring sounds. Thus, we modeled the snoring sounds source (Fig. 3) as a sinusoid signal with its frequency equal to the pitch of the recorded snoring sounds for every individual subject.

#### Subject-specific upper airway model

The upper airway was modeled as a collapsible tube^22^,^23^,^24^,^46^,^47^ (See supplementary Fig. S1). In this model, the air pressure and airflow were modeled as the voltage and current, respectively. The model included acoustic resistance of airflow (R_a_), compliance (C_a_), inertance (L_a_), and conductance (G_a_)^46^,^47^; as well as upper airway wall resistance (R_w_), inertance (L_w_) and compliance (C_w_)^22^,^24^,^48^. Detailed definitions of the model variables are presented in the supplementary file. In this study, we used the previously reported measurements for the air and tissue properties such as viscosity and elasticity^22^,^23^,^24^,^46^,^47^,^48^,^49^ (See supplementary Table S2). Previous models of the upper airway did not incorporate the differences in the upper airway anatomy among subjects such as its internal radius and wall thickness. For each subject, we used measurements of UA-XSA and NC to estimate the upper airway wall thickness (Fig. 3). Upper airway radius (T_r_) was calculated as square root of (UA-XSA/π), neck radius (N_r_) was estimated as (NC/2π), and the upper airway wall thickness (h) was calculated as (N_r_ − T_r_).

### Statistical analysis

The changes in NC, airway wall thickness, and UA-XSA from before to after sleep were assessed by paired t-tests for normally distributed data and Wilcoxon rank-sum test for non-normally distributed data. Paired t-tests were performed to investigate the differences between the measured average powers of recorded snoring sounds from the beginning to the end of sleep. The changes in measured snoring sounds features between different sleep stages were investigated by analysis of variance (ANOVA) and the post-hoc Tukey test. All the correlation analyses were performed based on Pearson or Spearman’s rank coefficient, for normally and non-normally distributed data, respectively. Furthermore, we performed Bland-Altman statistical test to verify the agreement between modeled and measured resonant frequencies. Statistical analyses were performed by Matlab and two-tailed P < 0.05 was considered as significant. Data are presented as mean ± SD.

## Additional Information

**How to cite this article**: Saha, S. *et al*. A Subject-Specific Acoustic Model of the Upper Airway for Snoring Sounds Generation. *Sci. Rep.* **6**, 25730; doi: 10.1038/srep25730 (2016).

## Supplementary Material

Supplementary Information

## Figures and Tables

**Table 1:** Characteristics of the participants (n = 20).

**Variable**	**Mean ± SD**
Age, years	45.1 ± 11.4
Height, cm	176.9 ± 6.3
Weight, kg	79.0 ± 10.7
Body Mass Index, kg/m^2^	25.4 ± 3.05
Neck circumference, cm	41.8 ± 2.9
Upper Airway Cross-Sectional Area, cm^2^	2.6 ± 0.6
Velum to Glottis length, cm	9.1 ± 1.8
Systolic Blood Pressure, mmHg	110.6 ± 8.5
Diastolic Blood Pressure, mmHg	76.0 ± 8.3

**Table 2:** Sleep structure (n = 20);

**Variable**	**Mean ± SD**
Total sleep time, min	150.1 ± 46.1
N1 sleep, %	18.0 ± 10.4
N2 sleep, %	57.2 ± 15.1
N3 sleep, %	11.5 ± 12.9
REM sleep, %	10.7 ± 8.1
Sleep efficiency, %	74.7 ± 15.0
Total AHI, /h	27.6 ± 25.8
Obstructive AHI, /h	25.5 ± 25.7
Central AHI, /h	2.0 ± 2.67

## References

[b1] Pevernagie D., Aarts R. M. & De Meyer M. The acoustics of snoring. Sleep Med. Rev. 14, 131–144 (2010). 19665907 10.1016/j.smrv.2009.06.002

[b2] Bloom J. W., Kaltenborn W. T. & Quan S. F. Risk factors in a general population for snoring. Importance of cigarette smoking and obesity. Chest 93, 678–683 (1988). 3258226 10.1378/chest.93.4.678

[b3] Huang L., Quinn S. J., Ellis P. D. & Williams J. E. Biomechanics of snoring. Endeavour 19, 96–100 (1995). 7493592 10.1016/0160-9327(95)97493-r

[b4] Dempsey J. A., Veasey S. C., Morgan B. J. & O’Donnell C. P. Pathophysiology of Sleep Apnea. Physiol. Rev. 90, 47–112 (2010). 20086074 10.1152/physrev.00043.2008 PMC3970937

[b5] White L. H. & Bradley T. D. Role of nocturnal rostral fluid shift in the pathogenesis of obstructive and central sleep apnoea. J. Physiol. (Lond.) 591, 1179–1193 (2013). 23230237 10.1113/jphysiol.2012.245159 PMC3607865

[b6] Verbraecken J. A. & De Backer, W. A. Upper airway mechanics. Respiration 78, 121–133 (2009). 19478479 10.1159/000222508 PMC2790795

[b7] Randerath W. J., Sanner B. M. & Somers V. K. Sleep apnea: current diagnosis and treatment. Vol. 35 (Karger Medical and Scientific Publishers, 2006).

[b8] Bliwise D. L., Nekich J. C. & Dement W. C. Relative validity of self-reported snoring as a symptom of sleep apnea in a sleep clinic population. Chest 99, 600–608 (1991). 1995215 10.1378/chest.99.3.600

[b9] Maimon N. & Hanly P. J. Does snoring intensity correlate with the severity of obstructive sleep apnea? J. Clin. Sleep Med. 6, 475–478 (2010). 20957849 PMC2952752

[b10] Nakano H., Furukawa T. & Nishima S. Relationship Between Snoring Sound Intensity and Sleepiness in Patients with Obstructive Sleep Apnea. J. Clin. Sleep Med. 4, 551–556 (2008). 19110884 PMC2603532

[b11] Fiz J. A. . Acoustic analysis of snoring sound in patients with simple snoring and obstructive sleep apnoea. Eur. Respir. J. 9, 2365–2370 (1996). 8947087 10.1183/09031936.96.09112365

[b12] Xu H., Huang W., Yu L. & Chen L. Sound spectral analysis of snoring sound and site of obstruction in obstructive sleep apnea syndrome. Acta Otolaryngol. 130, 1175–1179 (2010). 20377505 10.3109/00016481003694774

[b13] Ng A. K., San Koh T., Abeyratne U. R. & Puvanendran K. Investigation of obstructive sleep apnea using nonlinear mode interactions in nonstationary snore signals. Ann. Biomed. Eng. 37, 1796–1806 (2009). 19551511 10.1007/s10439-009-9744-8

[b14] Ng A. K. . Could formant frequencies of snore signals be an alternative means for the diagnosis of obstructive sleep apnea? Sleep Med. 9, 894–898 (2008). 17825609 10.1016/j.sleep.2007.07.010

[b15] Alencar A. M. . Dynamics of snoring sounds and its connection with obstructive sleep apnea. Phys. A: Statistical Mechanics and its Applications 392, 271–277 (2013).

[b16] Liu Z., Luo X., Lee H. & Lu C. Snoring source identification and snoring noise prediction. J. Biomech. 40, 861–870 (2007). 16737702 10.1016/j.jbiomech.2006.03.022

[b17] Ng A. K., Koh T. S., Baey E. & Puvanendran K. Role of upper airway dimensions in snore production: acoustical and perceptual findings. Ann. Biomed. Eng. 37, 1807–1817 (2009). 19551510 10.1007/s10439-009-9745-7

[b18] Yadollahi A. & Moussavi Z. M. A robust method for heart sounds localization using lung sounds entropy. IEEE Trans. Biomed. Eng. 53, 497–502 (2006). 16532776 10.1109/TBME.2005.869789

[b19] Pasterkamp H., Kraman S. S. & Wodicka G. R. Respiratory sounds. Advances beyond the stethoscope. Am. J. Respir. Crit. Care Med. 156, 974–987 (1997). 9310022 10.1164/ajrccm.156.3.9701115

[b20] Gavriely N., Shee T. R., Cugell D. W. & Grotberg J. B. Flutter in flow-limited collapsible tubes: a mechanism for generation of wheezes. J. Appl. Physiol. 66, 2251–2261 (1989). 2745288 10.1152/jappl.1989.66.5.2251

[b21] Boersma P. & KIRCHNER R. Functional Phonology. Formalizing the interactions between articulatory and perceptual drives. Glot international 4, 13–15 (1999).

[b22] Wodicka G. R., Stevens K. N., Golub H. L., Cravalho E. G. & Shannon D. C. A model of acoustic transmission in the respiratory system. IEEE Trans. Biomed. Eng. 36, 925–934 (1989). 2777281 10.1109/10.35301

[b23] Harper V. P., Pasterkamp H., Kiyokawa H. & Wodicka G. R. Modeling and measurement of flow effects on tracheal sounds. IEEE Trans. Biomed. Eng. 50, 1–10 (2003). 12617519 10.1109/TBME.2002.807327

[b24] Harper P., Kraman S. S., Pasterkamp H. & Wodicka G. R. An acoustic model of the respiratory tract. IEEE Trans. Biomed. Eng. 48, 543–550 (2001). 11341528 10.1109/10.918593

[b25] Finkelstein Y. . Velopharyngeal anatomy in patients with obstructive sleep apnea versus normal subjects. J. Oral Maxillofac. Surg. 72, 1350–1372 (2014). 24485981 10.1016/j.joms.2013.12.006

[b26] Maltais F., Carrier G., Cormier Y. & Sériès F. Cephalometric measurements in snorers, non-snorers, and patients with sleep apnoea. Thorax 46, 419–423 (1991). 1858079 10.1136/thx.46.6.419 PMC463188

[b27] Paliwal K. K. Spectral subband centroid features for speech recognition. in Proceedings of the IEEE International Conference on Acoustics, Speech and Signal Processing 617–620 (IEEE) (1998).

[b28] Beck R., Odeh M., Oliven A. & Gavriely N. The acoustic properties of snores. Eur. Respir. J. 8, 2120–2128 (1995). 8666109 10.1183/09031936.95.08122120

[b29] Gavriely N. & Jensen O. Theory and measurements of snores. J. Appl. Physiol. *(1985)* 74, 2828–2837 (1993). 8365987 10.1152/jappl.1993.74.6.2828

[b30] Sforza E. . Upper airway collapsibility and cephalometric variables in patients with obstructive sleep apnea. Am. J. Respir. Crit. Care Med. 161, 347–352 (2000). 10673170 10.1164/ajrccm.161.2.9810091

[b31] Fiz J. A. . Acoustic analysis of vowel emission in obstructive sleep apnea. Chest 104, 1093–1096 (1993). 8404173 10.1378/chest.104.4.1093

[b32] Robb M., Yates J. & Morgan E. Vocal tract resonance characteristics of adults with obstructive sleep apnea. Acta Otolaryngol. 117, 760–763 (1997). 9349877 10.3109/00016489709113474

[b33] Quinn S. J., Huang L., Ellis P. D. & Williams J. E. The differentiation of snoring mechanisms using sound analysis. Clin. Otolaryngol. Allied Sci. 21, 119–123 (1996). 8735394 10.1111/j.1365-2273.1996.tb01313.x

[b34] Yadollahi A. . A randomized, double crossover study to investigate the influence of saline infusion on sleep apnea severity in men. Sleep 37, 1699–1705 (2014). 25197812 10.5665/sleep.4084 PMC4173926

[b35] Clark S. A., Wilson C. R., Satoh M., Pegelow D. & Dempsey J. A. Assessment of inspiratory flow limitation invasively and noninvasively during sleep. Am. J. Respir. Crit. Care Med. 158, 713–722 (1998). 9730995 10.1164/ajrccm.158.3.9708056

[b36] Epstein L. J. . Clinical guideline for the evaluation, management and long-term care of obstructive sleep apnea in adults. J Clin Sleep Med 5.3, 263–276 (2009). 19960649 PMC2699173

[b37] Fredberg J. J., Wohl M. E., Glass G. M. & Dorkin H. L. Airway area by acoustic reflections measured at the mouth. J. Appl. Physiol. Respir. Environ. Exerc. Physiol. 48, 749–758 (1980). 7451282 10.1152/jappl.1980.48.5.749

[b38] Yadollahi A., Rudzicz F., Mahallati S., Coimbra M. & Bradley T. D. Acoustic estimation of neck fluid volume. Ann. Biomed. Eng. 42, 2132–2142 (2014). 25103604 10.1007/s10439-014-1083-8

[b39] Yadollahi A. & Moussavi Z. M. A robust method for estimating respiratory flow using tracheal sounds entropy. IEEE Trans. Biomed. Eng. 53, 662–668 (2006). 16602572 10.1109/TBME.2006.870231

[b40] Oppenheim A. V., Willsky A. S. & Nawab S. H. Signals and systems vol. 2. (Prentice-Hall Englewood Cliffs, NJ, 1983).

[b41] Klapuri A. & Davy M. Signal processing methods for music transcription. (Springer Science & Business Media, 2007).

[b42] Talkin D. A Robust Algorithm for Pitch Tracking (RAPT) in Speech coding and synthesis Ch. 14, 495–518 (Elsevier Sciences, 1995).

[b43] Kaniusas E. Linking physiological phenomena and biosignals. (Springer, 2012).

[b44] Deller J. R., Hansen J. H. L. & Proakis J. G. Discrete-time processing of speech signals. (Institute of Electrical and Electronics Engineers Press, 2000).

[b45] Voicebox Voicebox: Speech processing toolbox for matlab written by Brookes, M., Department of Electrical & Electronic Engineering, Imperial College, London, UK. URL http://www.ee.ic.ac.uk/hp/staff/dmb/voicebox/voicebox.html (1997).

[b46] Fant G. Acoustic Theory of Speech Production. (Mouton, 1970).

[b47] Flanagan J. L. Speech analysis, synthesis and perception. Vol. 3 (Springer Science & Business Media, 2013).

[b48] Habib R. H., Chalker R. B., Suki B. & Jackson A. C. Airway geometry and wall mechanical properties estimated from subglottal input impedance in humans. J. Appl. Physiol. 77, 441–451 (1994). 7961268 10.1152/jappl.1994.77.1.441

[b49] Mansfield J. & Wodicka G. Using acoustic reflectometry to determine breathing tube position and patency. J. Sound Vibration 188, 167–188 (1995).

