# Supplementary material for: A Subject-Specific Acoustic Model of the Upper Airway for Snoring Sounds Generation
Source: Sci Rep. 2016 May 23;6:25730. doi: 10.1038/srep25730 (PMC4876448; doi:10.1038/srep25730)
Supplement: Supplementary Information [file srep25730-s1.pdf]

Supplementary File

Title: A Subject-Specific Acoustic Model of the Upper Airway for Snoring Sounds Generation

Authors: Shumit Saha[1,2], T. Douglas Bradley[2,3], Mahsa Taheri[2], Zahra Moussavi[1], *Azadeh Yadollahi[2,4]

[1] Department of Biomedical Engineering, University of Manitoba;

[2] Toronto Rehabilitation Institute-University Health Network

[3] Department of Medicine, University of Toronto

[4] Institute of Biomaterials and Biomedical Engineering, University of Toronto

Contact Information:

Shumit Saha: sahas34@myumanitoba.ca

T. Douglas Bradley: douglas.bradley@utoronto.ca

Mahsa Taheri: mahsa.taheri87@gmail.com

Zahra Moussavi: Zahra.Moussavi@umanitoba.ca

Azadeh Yadollahi: Azadeh.Yadollahi@uhn.ca

Corresponding Author:

Azadeh Yadollahi, PhD
Scientist, University Health Network-Toronto Rehabilitation Institute
Assistant Professor, Institute of Biomaterials & Biomedical Engineering, University of Toronto
Email: Azadeh.Yadollahi@uhn.ca
Room 12-106, 550 University Ave., Toronto, ON., M5G 2A2
Phone (office): 416 597 3422 x 7936
Fax: 416 597 8959

Feature Extraction of Snoring Sounds:

We extracted several temporal and spectral features for all snoring segments (Table S1).

Since sleep stage may change the upper airway control mechanism and the generation of snoring sounds[1], we investigated the patterns of snoring occurrences for the entire sleep and every sleep stage separately. We calculated two features: snoring percentage, which represent the number of snoring segments in each sleep stage divided by the total number of snoring segments in the entire sleep; and snoring time index (STI), representing the total snoring time in each sleep stage divided by time spent in each sleep stage

For calculating spectral features, we first estimated power spectral density (PSD) using welch method with 100 ms hamming window and 50% overlap between adjacent windows. We calculated spectral features for the entire frequency band (100-4000 Hz), and seven sub-bands: 100-150 Hz, 150-450 Hz, 450-600 Hz, 600-1200 Hz, 1200-1800 Hz, 1800-2500 Hz, and 2500-4000 Hz [2],[3]. We choose these sub-bands to determine the dominant and the resonance frequency bands in snoring sounds. Frequencies below 100 Hz were not chosen to remove the effects of heart sounds as frequencies below 100 Hz often contaminated with them[4]. Previous studies have shown that the main frequencies of snoring due to the narrowing at the base of tongue were above 650 Hz; whereas frequencies of palatal snoring were below 450 Hz[5,6]. Fruthermore, previous studies have shown that most of the main peaks of snoring were less than 1000 Hz[7]. Additionally, in some cases it was also reported that the harmonics of the frequencies could be expanded to 2200 and 3500 Hz[8]. Also, the measured first and second formant frequencies (resonance frequencies) of snoring sounds are mostly in the range of 400-800 Hz, 1200 – 1800 Hz respectively[9,10]. Considering all these facts, we choose those seven sub-bands.

From PSD, we calculated three spectral features. The spectral features included the average power of snoring sounds in each frequency band; relative power, which defines as the average power of snoring segments in each sub-band divided by the average power in entire frequency band (100-4000 Hz); and spectral centroid, which determines the frequency with the maximum power in each frequency band.

Table S1: Features calculated from the snoring segments

**Feature Name**	**Equation/ Method**
Snoring Percentage, %	Snoring in Sleep stage/ Total number of snoring
Snoring Time Index, %	Total Snoring Time/ Total Sleeping Time
Average Signal Power, P_{avg} , dB*	$P_{avg}\ (f_l \leq f \leq f_u) = \sum_{(f_l \leq f \leq f_u)} p(f)\Delta f$
Relative Signal Power (RSP), %*	$\frac{P(f_l \leq f \leq f_u)}{P(100 \leq f \leq 4000)}$
Spectral Centroid (SC), Hz*	$\frac{\sum_{(f_l \leq f \leq f_u)} f p(f)\Delta f}{P_{avg}\ (f_l \leq f \leq f_u)}$
Pitch, Hz	Robust Algorithm for Pitch Tracking (RAPT)
Formant Frequencies, Hz	Linear Predictive Coding (LPC) analysis

$p(f)$ = estimated amplitude by power spectral density
f_l = Lower band frequency and f_u = Higher band frequency.
* Feature was computed over the entire frequency band: 100 – 4000Hz and seven sub-bands of the power spectrum: 100, 150; 150, 450; 450, 600; 600, 1200; 1200, 1800; 1800, 2500; 2500-4000 Hz.

Upper Airway Model:

We developed an electrical equivalent circuit of the upper airway tube considering it as a collapsible tube (Figure 1). We used the impedance type analogy to derive the transfer function of the circuit. The impedance type analogy yields the voltage current relationship of a circuit. The acoustical pressure drop of an element is represented by the voltage drop across the electrical element. For an applied acoustical pressure drop, there is volume of fluid movement

happens in response. This fluid flow is represented by the electrical current through the circuit. So, the acoustical impedance can be derived by the acoustical pressure divided by the fluid volume flow. As the sounds wave propagates along a lossy tube, it experiences the viscous and heat conduction losses and dissipates energy. These losses can be represented by the acoustical resistance (R_a), inertance (L_a), and compliance (C_a). The thermal losses at the boundaries and during compressions and expansions are represented by R_a. The mass of medium is presented by L_a. The expansion and compression ability of the fluid medium is represented by the C_a. The heat conduction on the wall is presented by conductance (G_a). To consider the effects of wall vibration, the wall resistance (R_w), wall inertance (L_w) and wall compliance (C_w) were included in the model. Table S2 shows the equations of the element used in the model.

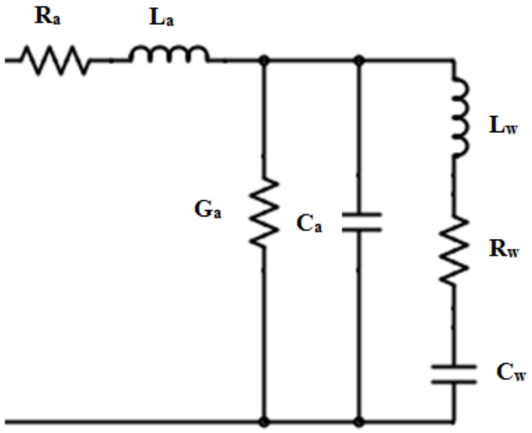

Figure S1: Electrical equivalent circuit of the upper airway

Table S2: Equation of the elements of the electrical circuit model of the upper airway (equations and values obtained from[11-17])

Element	Formula
Resistance	$Ra = \frac{2l}{\pi r^3}\sqrt{\frac{\omega \eta \rho_0}{2}}$
Inertance	$La = \frac{l\rho_0}{A}$

Compliance	$Ca = \frac{Al}{c^2\rho_0}$
Conductance	$Ga = 2\pi rl\frac{v-1}{c^2\rho_0}\sqrt{\frac{k\omega}{2\rho_0 c_p}}$
Wall Resistance	$Rw = \frac{\eta_w h}{2\pi lr^3}$
Wall Inertance	$Lw = \frac{\rho_w h}{2\pi rl}$
Wall Compliance	$Cw = \frac{2\pi lr^3}{E_w h}$

Density of Median (ρ_0): 1.14e-3 (Moist Air, 37 °C) g/cm^2; Shear Viscosity(η) : 1.86e-4 (20 Degree, 1 atm) dyne.s/ cm^2; Heat Conduction Coefficient (k): 0.064e-3 (37 °C) cal/cm-S-°C; Ratio of Specific Heat (v = c_p/c_v) :1.4; Specific Heat at Constant Pressure(c_p): 0.24 (0 °C, 1 atm) Cal/g-°C; Speed of Sounds(c): 3.54e4 (Moist Air, 37 °C) cm/s; Tissue Density(ρ_w): 1.06 g/ cm^2; Tissue Viscosity(η_w): 1.6e3 dyne.s/ cm^2; Tissue Elasticity(E_w): 0.392e6 dyne/ cm^2; ω: Radian Frequency; A: Cross Sectional Area; r: Tube Radius; l: Tube Length; h: Wall thickness.

References

1 Dempsey, J. A., Veasey, S. C., Morgan, B. J. & O'Donnell, C. P. Pathophysiology of Sleep Apnea. *Physiological Reviews* **90**, 47-112 (2010).

2 Yadollahi, A. & Moussavi, Z. M. A robust method for estimating respiratory flow using tracheal sounds entropy. *IEEE Trans Biomed Eng* **53**, 662-668, doi:10.1109/TBME.2006.870231 (2006).

3 Yadollahi, A., Rudzicz, F., Mahallati, S., Coimbra, M. & Bradley, T. D. Acoustic estimation of neck fluid volume. *Annals of Biomedical Engineering* **42**, 2132-2142, doi:10.1007/s10439-014-1083-8 (2014).

4 Yadollahi, A. & Moussavi, Z. M. A robust method for heart sounds localization using lung sounds entropy. *Biomedical Engineering, IEEE Transactions on* **53**, 497-502 (2006).

5 Quinn, S. J., Huang, L., Ellis, P. D. & Williams, J. E. The differentiation of snoring mechanisms using sound analysis. *Clin Otolaryngol Allied Sci* **21**, 119-123 (1996).

6 Xu, H., Huang, W., Yu, L. & Chen, L. Sound spectral analysis of snoring sound and site of obstruction in obstructive sleep apnea syndrome. *Acta oto-laryngologica* **130**, 1175-1179, doi:10.3109/00016481003694774 (2010).

7 Fiz, J. A. *et al.* Acoustic analysis of snoring sound in patients with simple snoring and obstructive sleep apnoea. *Eur. Respir. J.* **9**, 2365-2370 (1996).

8 Dalmasso, F. & Prota, R. Snoring: analysis, measurement, clinical implications and applications. *Eur. Respir. J.* **9**, 146-159 (1996).

9 Çavuşoğlu, M., Kamaşak, M., Çiloğlu, T., Serinağaoğlu, Y. & Eroğul, O. in *Proceedings of the Sixth IASTED International Conference on Biomedical Engineering.* 473-477 (ACTA Press).
10 Ng, A. K. *et al.* Could formant frequencies of snore signals be an alternative means for the diagnosis of obstructive sleep apnea? *Sleep Medicine* **9**, 894-898, doi:10.1016/j.sleep.2007.07.010 (2008).
11 Fant, G. *Acoustic Theory of Speech Production*. (Mouton, 1970).
12 Flanagan, J. L. *Speech analysis, synthesis and perception*. Vol. 3 (Springer Science & Business Media, 2013).
13 Mansfield, J. Theory and application of acoustic reflectometry in the human body. *Department of Electrical Engineering*, 208 (1996).
14 Wodicka, G. R., Stevens, K. N., Golub, H. L., Cravalho, E. G. & Shannon, D. C. A model of acoustic transmission in the respiratory system. *Biomedical Engineering, IEEE Transactions on* **36**, 925-934 (1989).
15 Habib, R. H., Chalker, R. B., Suki, B. & Jackson, A. C. Airway geometry and wall mechanical properties estimated from subglottal input impedance in humans. *Journal of Applied Physiology* **77**, 441-451 (1994).
16 Harper, P., Kraman, S. S., Pasterkamp, H. & Wodicka, G. R. An acoustic model of the respiratory tract. *IEEE Trans Biomed Eng* **48**, 543-550, doi:10.1109/10.918593 (2001).
17 Harper, V. P., Pasterkamp, H., Kiyokawa, H. & Wodicka, G. R. Modeling and measurement of flow effects on tracheal sounds. *IEEE Trans Biomed Eng* **50**, 1-10, doi:10.1109/TBME.2002.807327 (2003).
